# Association between self-reported sedentary behavior and health-related quality of life among infertile women with polycystic ovary syndrome

**DOI:** 10.1186/s12905-023-02222-5

**Published:** 2023-02-14

**Authors:** Yanjun Cao, Guopeng Li, Yanbei Ren

**Affiliations:** 1 Department of Neurology, The Second Hospital, Cheeloo College of Medicine, Shandong University, Jinan, 250012 Shandong People’s Republic of China; 2 Department of Health Psychology, School of Nursing and Rehabilitation, Cheeloo College of Medicine, Shandong University, Jinan, 250012 Shandong People’s Republic of China; 3 Department of Cardiology, Qilu Hospital of Shandong University, Jinan, 250012 Shandong People’s Republic of China

**Keywords:** Sedentary behavior, Physical activity, Anxiety, Depression, Quality of life, Polycystic ovary syndrome

## Abstract

**Background:**

High sedentary behavior and poor health-related quality of life (HRQoL) were common among women with polycystic ovary syndrome (PCOS). However, the association of sedentary behavior with HRQoL among infertile women with PCOS is still unknown. This study aimed to investigate the association of sedentary behavior with HRQoL among them.

**Methods:**

A cross-sectional study was conducted with 283 participants recruited from infertility outpatient clinic. A self-administered, structured questionnaire including the modified PCOS health-related QoL questionnaire (MPCOSQ), the International Physical Activity Questionnaire short form (IPAQ-SF), the Patient Health Questionnaire-9 (PHQ-9), and the Generalized Anxiety Disorder-7 (GAD-7) was used. Anthropometric and laboratory indictors related to PCOS were also collected. Multivariable linear regression analyses were performed to identify the associations. Bonferroni correction was utilized for multiple testing correction.

**Results:**

Sedentary behavior was associated with reduced HRQoL among this group. Specifically, over seven hours per day of sedentary behavior was strongly associated with total and several aspects of HRQoL (*β* ranged from − 0.378 to − 0.141, all *P* < 0.0063) after adjusting for physical activity, anxiety and depression. In addition, elevated BMI (*β* = − 0.407, *P* < 0.001) and anxiety (*β* ranged from − 0.410 to − 0.245, all *P* < 0.0063) were associated with poor HRQoL, while physical activity and depression were not.

**Conclusion:**

Sedentary behavior is an important behavior among infertile women with PCOS as it was associated with poorer HRQoL. Future interventions seeking to improve HRQoL should be considered to reduce sedentary behavior and psychological burden as primary intervention targets.

**Supplementary Information:**

The online version contains supplementary material available at 10.1186/s12905-023-02222-5.

## Introduction

Polycystic ovary syndrome (PCOS) is a chronic multisystem disorder with a high prevalence of 4–21% among reproductive age women [1, 2]. Clinical manifestations of PCOS encompass reproductive and metabolic abnormalities, such as hyperandrogenism, anovulation, infertility, insulin resistance, impaired glucose tolerance, type 2 diabetes and dyslipidemia [3]. The etiology of PCOS is still unclear; however, interactions between genetic and environmental factors and alterations in the microbiome have been postulated, involved in generating a hormonal and metabolic imbalance that can lead to the development of those gynecological problems and systemic metabolic syndrome [4]. Infertility is a potential concern among women with PCOS who want to conceive, and about 80% of infertility with anovulatory cycles can be attributed to PCOS [5]. Due to adverse impacts of infertility and other symptoms associated with PCOS, infertile women with PCOS consistently reported a higher risk of anxiety and depression [6–8]. Consequently, these factors contributed to a significant reduction in health-related quality of life (HRQoL) among infertile women with PCOS [9, 10].

To combat increased metabolic risks and improve the HRQoL associated with PCOS, lifestyle management targeting physical activity has been proposed to be first-line recommendation [3]. Significant improvements in body weight, metabolic features, reproductive, and HRQoL with a variety of lifestyle interventions targeting physical activity have been demonstrated [11, 12]. However, women with PCOS may experience more physical activity challenges because of individual-related barriers to weight and lifestyle management [13], lower rates of achievement in health goals [14] and high attrition rates in clinical weight management interventions [15]. In addition, adherence to an active physical lifestyle is poor and might decrease with weight gain [16, 17]. Thus, identifying additional modifiable behaviors with potential for promoting HRQoL and maintenance of function is warranted.

Sedentary behavior, an activity where the predominant posture is sitting or lying and low energy expenditure [18], may be one such set of modifiable behaviors. To perform moderate or vigorous intensity activity is a highly effortful, planned, volitional behavior, while to reduce sedentary behavior just need to get people up on their feet (if able) as often as possible [19]. Hence, it is reasonable to believe that interfering with sedentary behavior may be easier and more successful than interfering with physical activity, particularly for women with PCOS due to high rates of overweight and obesity [20]. Besides, A review of interventions in adults found that interventions that specifically targeted sedentary behavior rather than physical activity were more likely to reduce sedentary time and promote physical activity [21]. An increasing number of evidence implicated the adverse effects of sedentary behavior on physical health [22], mental health [23], disease incidence [24] and all-cause mortality [25]. Moreover, these deleterious health effects of sedentary behavior are different to those of physical inactivity and largely independent of an individual’s physical activity levels, which suggested that sedentary behavior is an independent predictor of physical and mental health [22, 26].

The research on the sedentary behavior of women with PCOS is still in the preliminary stage. Observational studies have shown that women with PCOS have higher sedentary time compared with women without PCOS [17, 27, 28], whilst the results of research on differences in physical activity are not consistent [17, 28]. Sedentary behavior had been shown to be strongly associated with weight gain and adverse metabolic profiles among women with PCOS [17, 29]. Frequently interrupting prolonged sitting with simple resistance activities could acutely improve endothelial function via increased resting shear rate and blood flow in women with PCOS [30]. However, to date, the association of sedentary behavior with HRQoL among infertile women with PCOS is still unknown. Moreover, studies on the relationship between sedentary behavior and HRQoL among non-PCOS individuals presented equivocal findings, such as few or no significant associations [31, 32]. Given the poorer HRQoL, high sedentary time and its potential health risks among infertile women with PCOS, understanding how these two outcomes are related may provide an additional therapeutic option for lifestyle intervention to improve HRQoL of infertile women with PCOS.

Therefore, this study aims to determine associations of sedentary time with HRQoL among infertile women with PCOS, accounting for the effects of physical activity levels, anxiety and depression. With studies showing the association between sedentary behaviors and weight gain and adverse metabolic profiles among women with PCOS [17, 29], it is hypothesized that higher amounts of sedentary time would be associated with poorer HRQoL among this group.

## Methods

### Participants and study design

A cross-sectional survey was conducted from infertility outpatient clinic at the Reproductive Medical Center of Shandong University between November 2020 and June 2021. The study was approved by the Research Ethics Committee of the affiliated institution. Informed consent was obtained from all individual participants included in the study. All infertile women with PCOS seeking their first treatment for infertility were consecutively recruited by a trained nurse. The inclusion criteria were as follows: (i) females aged more than 18, (ii) at least one year of infertility history, (iii) diagnosed with PCOS, and (iv) ability to understand and answer the questionnaires. PCOS was diagnosed by modified Rotterdam criteria [33], which included menstrual abnormalities (irregular uterine bleeding, oligomenorrhea, or amenorrhea) combined with either hyperandrogenism or polycystic ovaries. The exclusion criterion was having a history of mental illness or a severe medical condition that could interfere with study variable assessments. The sample size was estimated as 262, based on a relatively small effect size (*f*^2^) of 0.05 [31], α of 0.05, and power of 0.95 using G*Power [34]. Overall, 290 infertile women with PCOS voluntarily consented to participate in the study. After eliminating incomplete questionnaires, 283 participants were included in the final analysis.

### Measures

A self-administered, structured questionnaire including instruments for assessing socio-demographic and disease-related characteristics, anxiety, depression, physical activity and sedentary behavior, and HRQoL was used.

The socio-demographic and disease-related characteristics included age, education, monthly income, employment status, number of live born children, and duration of infertility.

The modified PCOS health-related QoL questionnaire (MPCOSQ) was used to assess health-related and disease-specific QoL among women experiencing PCOS problems [35]. It contains 30 items measuring the following 7 subscales: emotional disturbances (7 items), weight (6 items), hirsutism (5 items), acne (3 items), infertility (4 items), menstrual symptom (3 items), and menstrual predictability (2 items). Response categories are rated on a seven-point Likert scale ranging from 1 suggests maximum impairment to 7 indicates no problems or difficulties on that item. The average of the items scores are the total scores of each domain and MPCOSQ, with higher scores indicating better QoL. The Chinese version of the MPCOSQ had been verified with good reliability and validity [36]. In this study, the Cronbach’s alpha for the MPCOSQ was 0.939 and ranged between 0.728 and 0.954 for the domains.

The International Physical Activity Questionnaire short form (IPAQ-SF) was used to measures an individual’s physical activity and sedentary behavior in the last seven days [37]. It consists of seven items and collects information on time spent in walking, moderate-intensity PA, in vigorous-intensity PA and sitting. The first 6 items measure the frequency and duration of each activity during the last 7 days. The last item measures the duration of time spent sitting on a week day. Total weekly physical activity (MET-minutes/week) and its categorical levels were determinated by the scoring protocol. More than seven hours per day sitting time was defined as sedentary behavior as recommended by a previous meta-analysis [38].

The Patient Health Questionnaire-9 (PHQ-9) was used to assess the prevalence and levels of depression [39]. It consists of nine items rated each of the items ranging from 0 (not at all) to 3 (nearly every day). A score of 10 or greater represent for moderately to severe depression [40]. In this study, the Cronbach’s alpha was 0.887.

The Generalized Anxiety Disorder-7 (GAD-7) was used to assess the prevalence and levels of anxiety [41]. It consists of seven items rated each of the items ranging from 0 (not at all) to 3 (nearly every day). A score of 10 or greater represent for moderate to severe anxiety [40]. In this study, the Cronbach’s alpha was 0.950.

### Clinical variables

Body Height, Weight, Waist Circumference (WC) and Hip Circumference (HC) of participants were measured by a trained nurse according to standard protocols. BMI was computed as weight in kilograms divided by height in meters squared (kg/m^2^). Waist-to-hip ratio (WHR) was computed as the ratio of WC to HC. Results of laboratory tests including Anti-Mullerian hormone (AMH), total testosterone, sex hormone binding globulin (SHBG), fasting plasma glucose and fasting insulin levels were obtained from medical records. The homeostasis model assessment 2 (HOMA2‐IR) was calculated as a measure of insulin resistance (available at http://www.dtu.ox.ac.uk/homacalculator/).

### Statistical analysis

Data analysis was conducted by SPSS version 26.0 (IBM Corp., 2019). Descriptive statistics were used to characterize the data. Categorical measures were reported using frequency and percentage. Continuous measures were reported using mean, standard deviation (SD) or median, interquartile ranges (IQR) depending on distribution determinated by the Shapiro–Wilk test. Independent sample t test, One-Way ANOVA test and Pearson correlation were applied to determine the differences in MPCOSQ between the groups. Pearson and Spearman’s correlation coefficients were used to assess the correlation among depression, anxiety, physical activity, sedentary behavior, and MPCOSQ.

Finally, multivariate linear regression models were applied to examine associations of sedentary behavior and total and subscales of MPCOSQ. All models were adjusted for depression, anxiety, physical activity, and socio-demographic and clinical variables that were significantly associated with dependent variables in the univariate analyses. Statistical significance was defined as *P* < 0.05. Bonferroni correction was utilized for multiple testing correction (*P* < 0.05/8, outcome = 0.0063).

## Results

### Participants’ characteristics

The characteristics of the participants are summarized in Table 1. The mean age of 283 participants was 29.35 (SD = 4.19) years. The mean BMI was 27.02 (SD = 4.57) kg/m2. The mean WHR was 0.89 (SD = 0.068). 44.2% had completed at least a college education; 23.0% had a low average monthly household income (< ¥3000); and 73.1% were employed. The mean infertility duration was 3.62 (SD = 2.27), and 86.2% had no child. The mean levels of AMH, total testosterone, SHBG, fasting glucose, fasting insulin and HOMA2-IR were 9.82 (SD = 4.73) ng/ml, 43.99 (SD = 20.19) ng/dL, 34.40 (SD = 25.69) nmol/L, 5.72 (SD = 1.24) mmol/L, 20.72(SD = 10.73) μU/ml and 2.70 (SD = 1.38), respectively.

**Table 1:** Univariate analysis of total and subscales of MPCOSQ on Socio-demographic and clinical variables (n = 283)

Variables	N (%)/M ± SD	MPCOSQ	Emotional disturbance	Weight	Hirsutism	Acne	Infertility	Menstrual symptoms	Menstrual predictability
M ± SD		4.80 ± 0.93	4.56 ± 1.42	4.26 ± 1.37	5.69 ± 1.31	5.92 ± 1.32	3.63 ± 1.36	5.54 ± 1.12	3.49 ± 1.57
Age (years)	29.35 ± 4.19	0.119*	*0.90*	0.131*	*0.045*	*0.055*	*0.071*	*0.064*	*0.079*
Infertility duration(years)	3.62 ± 2.27	*0.010*	*−* *0.005*	*0.018*	*−* *0.020*	*0.010*	*−* *0.035*	*0.097*	*0.027*
BMI, kg/m^2^	27.02 ± 4.57	*−* 0.149*	*−* *0.032*	*−* 0.475**	*−* *0.022*	*0.065*	*−* *0.083*	*0.083*	*−* *0.021*
WHR	0.89 ± 0.07	*−* 0.119*	*−* *0.007*	*−* 0.254**	*−* *0.077*	*−* *0.077*	*−* *0.014*	*−* *0.095*	*0.001*
Educational		2.575*	3.049*	*2.049*	*0.846*	*0.788*	*1.319*	*0.493*	*1.453*
Less than junior high school	83(29.3)	4.95 ± 0.85	4.81 ± 1.37	4.51 ± 1.36	5.69 ± 1.42	6.04 ± 1.22	3.69 ± 1.29	5.61 ± 0.98	3.64 ± 1.51
Senior high school	75(26.5)	4.86 ± 1.05	4.66 ± 1.56	4.13 ± 1.50	5.84 ± 1.24	5.97 ± 1.29	3.79 ± 1.53	5.58 ± 1.31	3.61 ± 1.70
College or higher	125(44.2)	4.67 ± 0.89	4.34 ± 1.35	4.17 ± 1.41	5.59 ± 1.27	5.82 ± 1.40	3.48 ± 1.31	5.47 ± 1.82	3.31 ± 1.52
Monthly incomes (¥)		5.134**	3.796*	4.245*	*1.504*	*1.454*	*1.578*	*0.354*	5.80**
< 3000	65(23.0)	4.50 ± 0.96	4.14 ± 1.39	3.89 ± 1.35	5.45 ± 1.34	5.73 ± 1.53	3.37 ± 1.36	5.44 ± 1.30	3.01 ± 1.37
3000–6000	99 (35.0)	4.96 ± 0.79	4.68 ± 1.30	4.52 ± 1.40	5.79 ± 1.19	6.08 ± 1.07	3.75 ± 1.41	5.55 ± 1.04	3.84 ± 1.66
> 6000	119(42.0)	4.84 ± 0.98	4.69 ± 1.50	4.25 ± 1.31	5.73 ± 1.37	5.89 ± 1.38	3.66 ± 1.32	5.59 ± 1.08	3.44 ± 1.53
Employment status		*1.909*	2.097*	*2.118*	*0.296*	*0.446*	*1.814*	*0.971*	2.081*
Employed	250(73.1)	4.84 ± 0.90	4.63 ± 1.38	4.32 ± 1.34	5.70 ± 1.31	5.94 ± 1.29	3.68 ± 1.35	5.52 ± 1.12	3.56 ± 1.59
Unemployed	33(13.8)	4.51 ± 1.10	4.08 ± 1.66	3.79 ± 1.52	5.62 ± 1.32	5.83 ± 1.55	3.22 ± 1.45	5.72 ± 1.12	2.95 ± 1.34
Have child or not		*0.684*	*0.525*	*1.038*	*0.530*	*0.257*	*1.727*	*0.045*	*1.326*
Yes	39(13.8)	4.90 ± 0.88	4.67 ± 1.38	4.44 ± 1.38	5.58 ± 1.44	5.87 ± 1.12	3.97 ± 1.36	5.55 ± 1.06	3.79 ± 1.49
No	244(86.2)	4.79 ± 0.94	4.55 ± 1.43	4.23 ± 1.40	5.70 ± 1.29	5.93 ± 1.35	3.57 ± 1.36	5.54 ± 1.13	3.44 ± 1.58
AMH, ng/ml	9.82 ± 4.73	*−* *0.082*	*−* 0.121*	*0.109*	*−* 0.139*	*−* *0.051*	*−* *0.058*	*−* *0.051*	*−* 0.127*
Testosterone, ng/dL	43.99 ± 20.19	*−* *0.078*	*−* *0.079*	*−* *0.098*	*−* *0.058*	*0.047*	*−* *0.081*	*−* *0.004*	*−* *0.040*
SHBG, nmol/L	34.40 ± 25.69	0.161**	*0.053*	0.289**	0.128*	*0.056*	*0.066*	*−* *0.039*	*0.103*
Fasting glucose, mmol/L	5.72 ± 1.24	*0.018*	*0.050*	*−* *0.080*	*0.065*	*0.038*	*0.006*	*0.001*	*0.003*
Fasting insulin, μU/ml	20.72 ± 10.73	*−* 0.154**	*−* *0.094*	*−* 0.364**	*−* *0.083*	*0.025*	*−* *0.089*	*0.096*	*0.020*
HOMA2*−* IR	2.70 ± 1.38	*−* 0.143*	*−* *0.077*	*−* 0.364**	*−* *0.066*	*0.034*	*−* *0.085*	*0.093*	*0.021*

MPCOSQ modified polycystic ovary syndrome health-related quality-of-life questionnaire, BMI body mass index, WHR Waist-to-hip ratio, AMH anti-Mullerian Hormone, SHBG Sex hormone-binding globulin, HOMA2-IR homeostatic insulin resistance, M mean, SD Standard Deviation

*P < 0.05, ** *P* < 0.01, *** *P* < 0.001

Bold and italics highlights statistically significant after difference comparisons

### Univariate analyses

The results of univariate analyses were shown in Table 1. The mean score of MPCOSQ was 4.80 (SD = 0.93), with the scores of its seven subscales ranging from 3.49 to 5.54. The results of *t* test and One-Way ANOVA showed that the scores of total and subscales of MPCOSQ were significantly different between groups based on educational, monthly income, and employment. In addition, Pearson correlation analysis showed that total and subscales of MPCOSQ were significantly associated with age, BMI, WHR, AMH, SHBG, fasting insulin, and HOMA2-IR.

### Sedentary behavior, physical activity and MPCOSQ

The results of differences in total and subscales of MPCOSQ according to physical activity and sedentary behavior were presented in Table 2. The scores of MPCOSQ and its seven subscales were significant differences across sedentary time group (*t* ranged from 2.239 to 5.189, all *P* < 0.05). In addition, the scores of emotional disturbance and menstrual predictability were significant differences across the physical activity group (F = 2.232, 2.506, all *P* < 0.05).

**Table 2:** Total and subscales of MPCOSQ according to physical activity and Sedentary behavior. (n = 283)

Variables	N (%)/median (IQR)	MPCOSQ	Emotional disturbance	Weight	Hirsutism	Acne	Infertility	Menstrual symptoms	Menstrual predictability
Physical activity (MET.min/week)	458 (198, 1188)	*2.232*	2.237*	*1.044*	*1.197*	*1.145*	*1.317*	*0.474*	2.506*
Inactive	162 (57.2)	4.85 ± 0.97	4.61 ± 1.49	4.35 ± 1.43	5.77 ± 1.30	6.00 ± 1.30	3.64 ± 1.40	5.49 ± 1.12	3.44 ± 1.61
Moderate	97 (34.3)	4.66 ± 0.88	4.37 ± 1.28	4.10 ± 1.33	5.52 ± 1.35	5.76 ± 1.35	3.51 ± 1.36	5.58 ± 1.08	3.40 ± 1.46
Vigorous	24 (8.5)	5.06 ± 0.77	5.02 ± 1.37	4.33 ± 1.03	5.79 ± 1.19	6.07 ± 1.32	4.01 ± 1.07	5.71 ± 1.28	4.17 ± 1.65
Sedentary (h/d)	6(5, 8)	5.189***	3.988***	3.628***	3.373**	3.288**	2.954**	2.239*	3.482**
≤ 7 h/d	116(41.0)	5.03 ± 0.85	4.84 ± 1.34	4.50 ± 1.30	5.91 ± 1.09	6.14 ± 1.19	3.82 ± 1.36	5.66 ± 1.01	3.75 ± 1.61
> 7 h/d	167(59.0)	4.47 ± 0.94	4.17 ± 1.45	3.91 ± 1.39	5.36 ± 1.51	5.61 ± 1.44	3.34 ± 1.32	5.36 ± 1.24	3.10 ± 1.43

MPCOSQ modified polycystic ovary syndrome health-related quality-of-life questionnaire, IQR interquartile range

* *P* < 0.05, ** *P* < 0.01, *** *P* < 0.001

Bold and italics highlights statistically significant after difference comparisons

### Correlational analyses

Pearson and Spearman correlation results were showed in Table 3. MPCOSQ was negatively associated with depression (*r* = − 0.193, *P* < 0.01), anxiety (*r* = − 0.279, *P* < 0.001), and sedentary behavior (*r* = − 0.298, *P* < 0.001). The seven subscales of MPCOSQ were all significantly negatively associated with sedentary behavior (*r* ranged from − 0.232 to − 0.150, all *P* < 0.05). The six subscales of MPCOSQ were all significantly negatively associated with depression (*r* ranged from − 0.168 to − 0.131, all *P* < 0.05) and anxiety (*r* ranged from − 0.280 to − 0.122, all *P* < 0.05), except for the acne domain. In addition, sedentary behavior was negatively associated with physical activity (*r* = − 0.217, *P* < 0.001).

**Table 3:** Pearman and Spearman correlations of main study variables (n = 283)

Variables	1	1.1	1.2	1.3	1.4	1.5	1.6	1.7	2	3	4
1 MPCOSQ	1										
1.1 Emotional disturbance	0.839***	1									
1.2 Weight	0.777***	0.572***	1								
1.3 Hirsutism	0.613***	0.342***	0.272***	1							
1.4 Acne	0.559***	0.339***	0.258***	0.425***	1						
1.5 Infertility	0.726***	0.668***	0.626***	0.269***	0.129*	1					
1.6 Menstrual symptoms	0.475***	0.257***	0.274***	0.290***	0.247**	0.235**	1				
1.7 Menstrual predictability	0.518***	0.355***	0.365***	0.191**	0.212***	0.354***	0.288***	1			
2 PHQ-9	− 0.193**	− 0.168**	− 0.131*	− 0.077	− 0.089	− 0.162**	− 0.148*	− 0.160**	1		
3 GAD-7	− 0.279***	− 0.259***	− 0.189**	− 0.122*	− 0.093	− 0.280***	− 0.202**	− 0.168**	0.802***	1	
4 Physical activity^a^	0.010	0.024	− 0.013	− 0.055	− 0.055	0.038	0.106	0.077	− 0.020	− 0.011	1
5 Sedentary behavior^a^	− 0.298***	− 0.219***	− 0.221***	− 0.179**	− 0.232***	− 0.175**	− 0.150*	− 0.177**	0.116	0.186**	− 0.217***

MPCOSQ modified polycystic ovary syndrome health-related quality-of-life questionnaire, PHQ-9 Patient Health Questionnaire-9, GAD-7 Generalized Anxiety Disorder-7

^a^Spearman correlation

*P < 0.05, **P < 0.01, ***P < 0.001

### Multivariable linear regression analysis

The results of multivariable linear regression analysis for associations of sedentary behavior with total and subscales of MPCOSQ were presented in Table 4. The full models examining the correlates of total MPCOSQ and its seven subscales that displays all control variables could be found in Additional file 1: Supplementary Table 1. Results indicated that sedentary behavior was a significantly predictor for MPCOSQ (*β* = − 0.236, *P* < *0.001*), as well as five subscales of MPCOSQ (*β* ranged from − 0.378 to − 0.141, all *P* < 0.0063), except for the domain of infertility and menstrual symptoms. In addition, anxiety was a significantly predictor for MPCOSQ (*β* = − 0.330, *P* < *0.001*), as well as three subscales of MPCOSQ including emotional disturbance, weight and infertility (*β* = − 0.334, − 0.245, − 0.410, all *P* < 0.0063). BMI was a significantly predictor for weight domain (*β* = − 0.407, *P* < 0.001).

**Table 4:** Multivariable liner Regression analysis for associations of Sedentary behavior with total and subscales of MPCOSQ (n = 283)

Variables		B	SE	β	t	95%CI	P
Dependent	Independent						
MPCOSQ	GAD-7	− 0.067	0.019	− 0.330	− 3.618	− 0.104 to − 0.031	** < 0.001**
	Sedentary (> 7 h/d)	− 0.445	0.108	− 0.236	− 4.122	− 0.657 to − 0.232	** < 0.001**
Emotional disturbance	GAD-7	− 0.104	.029	− 0.334	− 3.537	− 0.161 to − 0.046	** < 0.001**
	Sedentary (> 7 h/d)	− 0.533	0.169	− 0.185	− 3.158	− 0.866 to − 0.201	**0.002**
Weight	Age	0.038	0.017	0.118	2.305	0.006 to 0.071	0.022
	BMI	− 0.122	0.020	− 0.407	− 5.995	− 0.161 to − 0.082	** < 0.001**
	GAD-7	− 0.073	0.025	− 0.245	− 2.939	− 0.122 to − 0.024	**0.004**
	Sedentary (> 7 h/d)	− 0.418	0.143	− 0.151	− 2.930	− 0.699 to − 0.137	**0.004**
Hirsutism	AMH	− 0.040	0.016	− 0.145	− 2.525	− 0.071 to − 0.009	0.012
	SHBG	0.007	0.003	0.132	2.305	0.001 to 0.012	0.022
	Sedentary (> 7 h/d)	− 0.549	0.153	− 0.207	− 3.583	− 0.851 to − 0.248	** < 0.001**
Acne	Sedentary (> 7 h/d)	− 0.535	0.158	− 0.200	− 3.376	− 0.847 to − 0.223	**0.001**
Infertility	GAD-7	− 0.122	.028	− 0.410	− 4.315	− 0.178 to − 0.066	** < 0.001**
	Sedentary (> 7 h/d)	− 0.407	0.158	− 0.147	− 2.573	− 0.718 to − 0.096	0.011
Menstrual symptoms	GAD-7	− 0.056	0.024	− 0.228	− 2.325	− 0.103 to − 0.009	0.021
	Sedentary (> 7 h/d)	− 0.250	0.134	− 0.110	− 1.868	− 0.513 to 0.014	0.063
Menstrual predictability	AMH	− 0.040	0.019	− 0.120	− 2.079	− 0.077 to − 0.002	0.039
	Sedentary (> 7 h/d)	− 0.569	0.188	− 0.179	− 3.024	− 0.940 to − 0.199	**0.003**

MPCOSQ modified polycystic ovary syndrome health-related quality-of-life questionnaire, GAD-7 Generalized Anxiety Disorder-7, BMI body mass index, AMH anti-Mullerian Hormone, SHBG Sex hormone-binding globulin, SE standard error, CI confidence interval

All models were controlled for depression (PHQ-9), anxiety (GAD-7), physical activity, and socio-demographic and clinical variables that were significantly associated with dependent variables in the univariate analyses. Only those control variables with a statistically significance before Bonferroni correction were presented in this table

Bold print highlights statistically significant after Bonferroni correction (*P* < 0.0063)

## Discussion

This study examined the association of sedentary behavior with HRQoL among infertile women with PCOS. The main finding was that self-reported sedentary behavior was negatively associated with HRQoL among this group. Specifically, over seven hours per day of sedentary behavior was significantly related to reduced total and several aspects of HRQoL, including emotional disturbance, physical (weight, hirsutism, acne), and menstrual predictability. Moreover, after adjusting for the potential confounders such as disease-specific biochemical indicators, physical activity, anxiety and depression, the association between sedentary behavior and HRQoL was still significant. In addition, this study found that elevated BMI and anxiety were associated with poor HRQoL among infertile women with PCOS, while physical activity and depression could not.

As hypothesized, the results revealed that sedentary behavior was associated with poor HRQoL among infertile women with PCOS, which were similar to previous results showing negative associations between sedentary behavior and HRQoL among individuals with cancer [42], Parkinson [43] and multimorbidity [44]. Several mechanisms might explain this relationship between sedentary behavior and HRQoL among infertile women with PCOS. From biological and functional perspective, engaging in prolonged periods of sitting behavior increases the likelihood of metabolic dysfunction, that plays an important role in the pathogenesis of PCOS and exacerbates the symptoms of PCOS that in turn may affect perceptions of own functioning and HRQoL [45, 46]. Indeed, there was significant difference in HOMA2-IR across sedentary time groups in this study (2.56 ± 1.29 Vs 2.91 ± 1.47, t = 2.170, *P* = 0.031). Compared with individuals who spent less than seven hours per day of sedentary behavior, infertile women with PCOS who spent at least seven hours of sedentary behavior had higher HOMA2-IR (2.91 ± 1.47), which indicated that sedentary behavior was related to increased metabolic risks among infertile women with PCOS. Alternatively, from psychosocial perspective, engaging in prolonged sedentary behaviors may lead to social solitude and withdrawing from interpersonal relationships which has been linked to increased social anxiety, thereby exacerbates psychiatric disorders and contributes to poor HRQoL among infertile women with PCOS [47, 48]. Moreover, this study showed that sedentary time was associated with an increased risk of anxiety, which could provide additional explanations.

This study demonstrated that physical activity had limited relationships with HRQoL among infertile women with PCOS. Although significantly difference in two domains of HRQoL including emotional disturbance and menstrual predictability between physical activity groups was found, physical activity did not significantly predict the total and domains of HRQoL after adjusting for sedentary behaviors and other related variables. In fact, most (91.5%) of participants had physical inactivity or moderate intensity of physical activity in this study, which indicated a lower level of physical active in this group and was comparable to study by Farnaz et al. (90.8%) [49]. Greenwood et al. found that vigorous but not moderate activity is associated with reduced odds of the metabolic syndrome in women with PCOS [50]. Therefore, the possible explanation of limited relationship between physical activity and HRQoL was that health benefits of lower levels of physical activity were insufficient to ameliorate the outcome of metabolic dysfunction, such as overweight or obesity, hirsutism and acne, which could not affect disease-specific HRQoL among infertile women with PCOS. Thus, further study of the development and maintenance of physical active behaviors among women with PCOS is warranted.

In addition, this study indicated that elevated BMI and anxiety were associated with poor HRQoL, which was in line with previous studies [51, 52]. Elevated BMI is associated with not only increased risk of metabolic disorders [53], but also lower body satisfaction and body image among women with PCOS [54], and consequently a poor perception of weight domain of HRQoL. Also, it was worth noting that no such relationship existed between BMI and the other domain of HRQoL, which suggesting obesity was not the only determinant of a poor HRQoL [51]. A systematic review had concluded that women with PCOS and concurrent anxiety had significant features of PCOS [7], such as higher mean values of BMI, hirsutism score and free testosterone, which linked to a reduced HRQoL [51]. Given that emotion disturbance is an important domain of HRQoL, it is expected that there would be a bidirectional association between anxiety and deteriorating HRQoL.

However, it should be mentioned that depression was found to be not associated with total and several domains of HRQoL in final controlled models, which was contrary to previous studies suggested that depression was significant predictor of reduced HRQoL related to PCOS [55]. Although Ye et al. found that anxiety but not depression was the stronger independent predictor of HRQoL in patients with obstructive sleep apnoea [56], there were no more published studies that have explored the independent contributions of anxiety and depression to HRQoL among women with PCOS. It was plausible that the different nature of anxiety and depression [57] or higher prevalence of depression than anxiety in this sample resulted in different contributions. Regarding small sample size in this study, it seems reasonable to avoid over-interpreting this result. Thus, future studies with a larger sample should be conducted to explore those relationships as high prevalence of moderate and severe depressive and anxiety symptoms among women with PCOS [7].

To our knowledge, this is the first study to examine association of sedentary behavior with HRQoL among infertile women with PCOS. Meanwhile, several potential confounders such as physical activity, anxiety and depression, and disease-specific biochemical indicators were controlled. These findings highlighted the interventions aimed at reducing sedentary behavior and psychological burden to improve HRQoL among infertile women with PCOS. In fact, empirical studies have confirmed the effectiveness of health education targeting on physical activities on lifestyle changes [58] and the effectiveness of cognitive-behavioral therapy on anxiety and depression [59] among patients with PCOS, which could be considered in future intervention studies. Despite these Strengths, several limitations should be considered when interpreting the results of this study. First, Sedentary behavior was assessed by one self-reported question form the IPAQ-SF, which were subjected to recall bias and limited the understanding of various sitting behaviors, such as occupational, leisure, transport. Thus, both device-based measures and instrument that assess sitting behaviors should be used. Second, due to the cross-sectional study design and small sample, the causal inference among variables and generalizability of the results are limited. Longitudinal studies with larger multi-center sample size are warranted. Last, the assessments were conducted based on participants’ self-reporting, which might be subjected to information bias.

## Conclusion

Overall, this study showed that sedentary behavior was strongly associated with poor several domains of HRQoL among infertile women with PCOS, after adjusting for physical activity, anxiety and depression, and disease-specific biochemical indicators. In addition, elevated BMI and anxiety were associated with poor HRQoL. Thus, our results verified that sedentary behavior was an important behavior to consider for promoting HRQoL. Future interventions seeking to improve HRQoL among infertile women with PCOS should be considered to reduce sedentary behavior and psychological burden.

## Supplementary Information

**Additional file 1: Supplemental Table 1.** Multivariable liner Regression analysis for associations of Sedentary behavior with total and subscales of MPCOSQ (n = 283).

## Data Availability

The datasets generated during and analyzed during the current study are available from the corresponding author on reasonable request.
