# Supplementary material for: Association between self-reported sedentary behavior and health-related quality of life among infertile women with polycystic ovary syndrome
Source: BMC Womens Health. 2023 Feb 14;23:67. doi: 10.1186/s12905-023-02222-5 (PMC9926864; doi:10.1186/s12905-023-02222-5)
Supplement: Supplementary file 1 — Additional file 1: Supplemental Table 1. Multivariable liner Regression analysis for associations of Sedentary behavior with total and subscales of MPCOSQ (n = 283). [file 12905_2023_2222_MOESM1_ESM.docx]

**Supplemental Table 1**

**S.Table 1 Multivariable liner Regression analysis for associations of Sedentary behavior with total and subscales of MPCOSQ (n=283)**

| **Variables** | | **B** | **SE** | **β** | **t** | **95%CI** | **P** |
| --- | --- | --- | --- | --- | --- | --- | --- |
| **Dependent** | **Independent** |  |  |  |  |  |  |
| **MPCOSQ** | Age | 0.022 | 0.012 | 0.098 | 1.752 | -0.003 to 0.046 | 0.081 |
|  | BMI | -0.021 | 0.015 | -0.105 | -1.404 | -0.051 to 0.009 | 0.161 |
|  | WHR | -0.594 | 0.789 | -0.046 | -0.753 | -2.148 to 0.959 | 0.452 |
|  | Educational | -0.055 | 0.067 | -0.050 | -0.825 | -0.187 to 0.077 | 0.410 |
|  | Income | -0.102 | 0.071 | -0.086 | -1.427 | -0.242 to 0.039 | 0.155 |
|  | SHBG | 0.004 | 0.002 | 0.110 | 1.802 | -0.001 to 0.008 | 0.073 |
|  | HOME2-IR | 0.005 | 0.048 | 0.008 | 0.110 | -0.090 to 0.101 | 0.913 |
|  | PHQ-9 | 0.017 | 0.017 | 0.093 | 1.018 | -0.016 to 0.050 | 0.310 |
|  | GAD-7 | -0.067 | 0.019 | -0.330 | -3.618 | -0.104 to -0.031 | **<0.001** |
|  | Physical activity | 0.011 | 0.080 | 0.008 | 0.137 | -0.147 to 0.169 | 0.891 |
|  | Sedentary (>7 h/d) | -0.445 | 0.108 | -0.236 | -4.122 | -0.657 to -0.232 | **<0.001** |
| **Emotional disturbance** | Educational | -0.089 | 0.106 | -0.053 | -0.841 | -0.297 to 0.119 | .401 |
|  | Income | -0.095 | .132 | -0.053 | -0.724 | -0.354 to 0.164 | .470 |
|  | Employment | 0.260 | .298 | 0.059 | 0.873 | -0.326 to 0.846 | .384 |
|  | AMH | -0.032 | .017 | -0.105 | -1.872 | -0.065 to 0.002 | .062 |
|  | PHQ-9 | 0.034 | .027 | 0.121 | 1.283 | -0.018 to 0.086 | .201 |
|  | GAD-7 | -0.104 | .029 | -0.334 | -3.537 | -0.161 to -0.046 | **<0.001** |
|  | Physical activity | 0.039 | .125 | 0.018 | 0.313 | -0.208 to 0.286 | .754 |
|  | Sedentary (>7 h/d) | -0.533 | 0.169 | -0.185 | -3.158 | -0.866 to -0.201 | **0.002** |
| **Weight** | Age | 0.038 | 0.017 | 0.118 | 2.305 | 0.006 to 0.071 | **0.022** |
|  | BMI | -0.122 | 0.020 | -0.407 | -5.995 | -0.161 to -0.082 | **<0.001** |
|  | WHR | -0.877 | 1.059 | -0.046 | -0.828 | -2.962 to 1.209 | 0.409 |
|  | Income | -0.170 | 0.089 | -0.098 | -1.913 | -0.346 to 0.005 | 0.057 |
|  | SHBG | 0.005 | 0.003 | 0.090 | 1.605 | -0.001 to 0.011 | 0.110 |
|  | HOME2-IR | -0.039 | 0.065 | -0.039 | -0.595 | -0.167 to 0.089 | 0.552 |
|  | PHQ-9 | 0.019 | 0.023 | 0.069 | 0.830 | -0.026 to 0.063 | 0.407 |
|  | GAD-7 | -0.073 | 0.025 | -0.245 | -2.939 | -0.122 to -0.024 | **0.004** |
|  | Physical activity | -0.059 | 0.107 | -0.028 | -0.548 | -0.270 to 0.152 | 0.584 |
|  | Sedentary (>7 h/d) | -0.418 | 0.143 | -0.151 | -2.930 | -0.699 to -0.137 | **0.004** |
| **Hirsutism** | AMH | -0.040 | 0.016 | -0.145 | -2.525 | -0.071 to -0.009 | **0.012** |
|  | SHBG | 0.007 | 0.003 | 0.132 | 2.305 | 0.001 to 0.012 | **0.022** |
|  | PHQ-9 | 0.011 | 0.025 | 0.042 | 0.441 | -0.038 to 0.060 | 0.660 |
|  | GAD-7 | -0.039 | 0.027 | -0.137 | -1.428 | -0.093 to 0.015 | 0.154 |
|  | Physical activity | -0.123 | 0.116 | -0.061 | -1.059 | -0.352 to 0.106 | 0.290 |
|  | Sedentary (>7 h/d) | -0.549 | 0.153 | -0.207 | -3.583 | -0.851 to -0.248 | **<0.001** |
| **Acne** | PHQ-9 | -0.014 | 0.026 | -0.052 | -0.526 | -0.064 to 0.037 | 0.599 |
|  | GAD-7 | -0.010 | 0.028 | -0.033 | -0.338 | -0.065 to 0.046 | 0.735 |
|  | Physical activity | -0.120 | 0.120 | -0.059 | -0.997 | -0.357 to 0.117 | 0.320 |
|  | Sedentary (>7 h/d) | -0.535 | 0.158 | -0.200 | -3.376 | -0.847 to -0.223 | **0.001** |
| **Infertility** | PHQ-9 | 0.048 | 0.026 | 0.176 | 1.860 | -0.003 to 0.098 | 0.064 |
|  | GAD-7 | -0.122 | .028 | -0.410 | -4.315 | -0.178 to -0.066 | **<0.001** |
|  | Physical activity | 0.057 | 00.120 | 0.027 | 0.479 | -0.179 to 0.294 | 0.633 |
|  | Sedentary (>7 h/d) | -0.407 | 0.158 | -0.147 | -2.573 | -0.718 to -0.096 | **0.011** |
| **Menstrual symptoms** | PHQ-9 | 0.009 | 0.022 | 0.042 | 0.434 | -0.033 to 0.052 | 0.665 |
|  | GAD-7 | -0.056 | 0.024 | -0.228 | -2.325 | -0.103 to -0.009 | **0.021** |
|  | Physical activity | 0.088 | 0.101 | 0.051 | 0.865 | -0.112 to 0.287 | 0.388 |
|  | Sedentary (>7 h/d) | -0.250 | 0.134 | -0.110 | -1.868 | -0.513 to 0.014 | 0.063 |
| **Menstrual predictability** | Income | 0.033 | 0.140 | 0.016 | 0.233 | -0.243 to 0.308 | 0.816 |
|  | Employment | 0.498 | 0.337 | 0.102 | 1.478 | -0.165 to 1.162 | 0.141 |
|  | AMH | -0.040 | 0.019 | -0.120 | -2.079 | -0.077 to -0.002 | **0.039** |
|  | PHQ-9 | -0.018 | 0.030 | -0.058 | -0.605 | -0.077 to 0.041 | 0.545 |
|  | GAD-7 | -0.035 | 0.033 | -0.101 | -1.044 | -0.100 to 0.031 | 0.297 |
|  | Physical activity | 0.168 | 00.141 | 0.070 | 1.193 | -0.109 to 0.446 | 0.234 |
|  | Sedentary (>7 h/d) | -0.569 | 0.188 | -0.179 | -3.024 | -0.940 to -0.199 | **0.003** |

MPCOSQ modified polycystic ovary syndrome health-related quality-of-life questionnaire, PHQ-9 Patient Health Questionnaire-9, GAD-7 Generalized Anxiety Disorder-7, BMI body mass index, WHR Waist-to-hip ratio, AMH anti-Mullerian Hormone, SHBG Sex hormone-binding globulin, HOMA2-IR homeostatic insulin resistance, M mean, SD Standard Deviation, SE standard error, CI confidence interval.

Bold print highlights statistically significant (P<0.05).
